# Controllable Synthesis of TiO_2_@Fe_2_O_3_ Core-Shell Nanotube Arrays with Double-Wall Coating as Superb Lithium-Ion Battery Anodes

**DOI:** 10.1038/srep40927

**Published:** 2017-01-18

**Authors:** Yan Zhong, Yifan Ma, Qiubo Guo, Jiaqi Liu, Yadong Wang, Mei Yang, Hui Xia

**Affiliations:** 1 School of Materials Science and Engineering, Nanjing University of Science and Technology, Nanjing 210094, China; 2 Herbert Gleiter Institute of Nanoscience, Nanjing University of Science and Technology, Nanjing 210094, China; 3 School of Engineering, Nanyang Polytechnic, 569830, Singapore

## Abstract

Highlighted by the safe operation and stable performances, titanium oxides (TiO_2_) are deemed as promising candidates for next generation lithium-ion batteries (LIBs). However, the pervasively low capacity is casting shadow on desirable electrochemical behaviors and obscuring their practical applications. In this work, we reported a unique template-assisted and two-step atomic layer deposition (ALD) method to achieve TiO_2_@Fe_2_O_3_ core-shell nanotube arrays with hollow interior and double-wall coating. The as-prepared architecture combines both merits of the high specific capacity of Fe_2_O_3_ and structural stability of TiO_2_ backbone. Owing to the nanotubular structural advantages integrating facile strain relaxation as well as rapid ion and electron transport, the TiO_2_@Fe_2_O_3_ nanotube arrays with a high mass loading of Fe_2_O_3_ attained desirable capacity of ~520 mA h g^−1^, exhibiting both good rate capability under uprated current density of 10 A g^−1^ and especially enhanced cycle stability (~450 mA h g^−1^ after 600 cycles), outclassing most reported TiO_2_@metal oxide composites. The results not only provide a new avenue for hybrid core-shell nanotube formation, but also offer an insight for rational design of advanced electrode materials for LIBs.

Lithium-ion batteries (LIBs) have been regarding as predominant power sources due to their excellent power-energy reliability, long cycle life and environmental benignity^1^,^2^,^3^. However, to date, their performance still lags behind the development of emerging applications, such as electric vehicles and multifunctional portable electronics, which still remains as the major hurdle for its large scale implementation in the future. As a vital part of LIBs, anode materials cast great influence on overall performance and need to be equipped with higher capacity, better rate capability and outstanding cycle ability^4^,^5^. In this sense, conventional graphite electrodes are trapped in low specific capacity (372 mA h g^−1^, as well as other issues, such as inferior power delivery and potential safety hazards resulted from low operating voltage (below 0. 2 V versus Li/Li^+^)^6^,^7^. Nano-scaled metal oxides (SnO_2_, TiO_2_, Fe_2_O_3_, Fe_3_O_4_, etc.) hold great promise as available alternative anode materials^8^,^9^, exhibiting relatively high specific capacity, nontoxicity, high corrosion resistance, low-cost processing and simplicity of syntheses^10^,^11^.

Amongst all, TiO_2_-based materials attracted a lot of attention and stimulated extensive researches based on their excellent merits serving as LIBs anodes. TiO_2_ possesses safe and stable working plateau potential (about 1.5–1.75 V versus Li/Li^+^) without intense decomposition of electrolyte. Furthermore, taking advantage of favorable crystallographic characteristics and surface activity with negligible volume change during charging/discharging processes, TiO_2_ material shows good structural stability, stable voltage output and long lifespan^12^,^13^,^14^. Nevertheless, it is regretted that TiO_2_ only delivers a low theoretical capacity (only 335 mA h g^−1^), which is even inferior to graphite^15^,^16^. On the other hand, as an another family member of transition metal oxides, Fe_2_O_3_ is endowed a theoretical capacity as high as 1005 mA h g^−1^, showing great prospect towards high energy anodes^11^. Frustratingly, accompanying the multi-electron reaction, concomitant repeating formation of metal and Li_2_O matrix will lead to dramatic volume variation and finally result in electrode pulverization and drastic capacity fading^17^,^18^. Apparently, the complementary features of these two oxides allow artful design of corresponding hybrid structures and lead to large numbers of delicate fabrication focusing TiO_2_ and Fe_2_O_3_ smart nanostructures^15^,^19^.

It is acknowledged that hybrid TiO_2_ with high-capacity metal oxides or carbon matrix is an efficient route to improve the electrochemical performance^20^,^21^,^22^. Till now, various nanostructured TiO_2_/Fe_2_O_3_ composites are taken up into anode materials^23^,^24^,^25^, showing improvement on capacity as well as upgrading on cycling stability. However, in most cases, the hybrid structures usually deliver unsatisfying performance than expected, especially for deficient rate ability and low capacity, which are mainly ascribed to insufficient contact at interfaces, large share of carbon constituent as conductive and stuffed component with long transport distance. As summarized, most literatures on TiO_2_@metal oxides as anodes for LIBs, e.g. TiO_2_@MnO_2_ nanowire array, TiO_2_@Fe_2_O_3_ nanorod array, and TiO_2_@Co_3_O_4_ nanobelt array, were normally tested at low current densities under 2.0 A/g, and exhibited depressing capacities below 300 mA h g^−1^ once the current density reaches over 1.6 A/g^25^. Moreover, uncontrollable synthesis makes it hard to achieve optimal balance in the synergistic effect. Herein, we present a binder-additive-free TiO_2_/Fe_2_O_3_ core-shell nanotubular arrays as high performance electrode through a unique method combining hydrothermal and stepwise atomic layer deposition (ALD). First of all, this 3D nanotubular architecture leads to a much larger surface area with adequate electrolyte penetration and direct 1D pathway for electron transport with also neighboring space to accommodate volumetric change of electrode materials^3^,^26^,^27^. Compared with traditional array structure, this core-shell nanotube design is further vested with friendly interface between components along with continuously intimate contact, more efficient ion and electron transport within hollow arrays as well as double-side area for high loading of Fe_2_O_3_^28^,^29^. Particularly, ADL facilitates facile construction of Fe_2_O_3_/TiO_2_ interface and controllable loading to investigate synergistic effects^30^,^31^. With the optimal effect of Fe_2_O_3_ coating, hybrid electrode exhibits outstanding electrochemical performance with especially outstanding cycle stability (~450 mA h g^−1^ after 600 cycles) and superb rate capability (up to 10 A g^−1^ charging), demonstrating great potential as excellent anode alternative for high performance LIBs.

## Results

**Figure 1:**
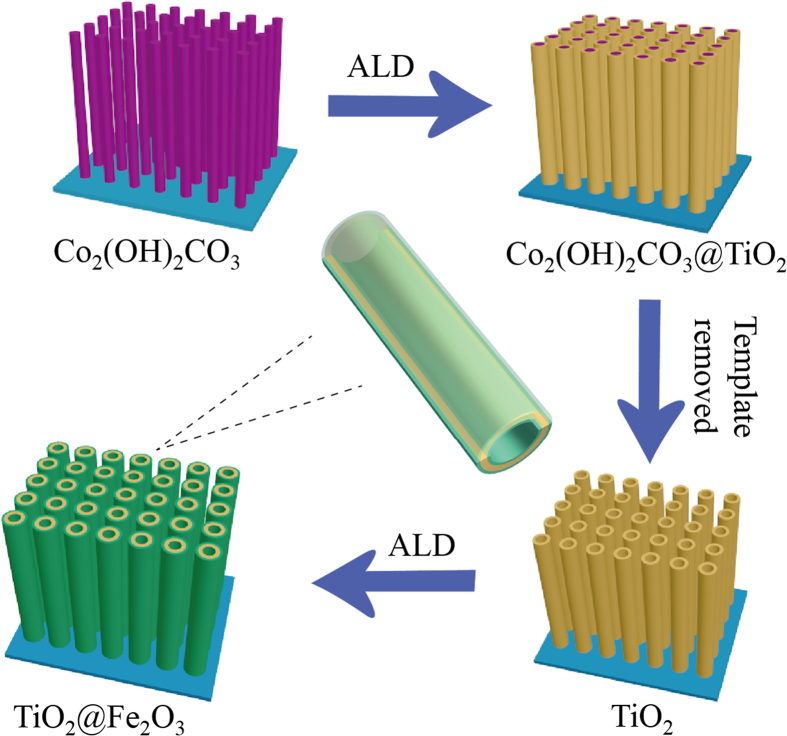
Schematic illustration of the formation process for the TiO_2_@Fe_2_O_3_ core-shell nanotube arrays with double-side coating.

The fabrication process of hollow TiO_2_@Fe_2_O_3_ core/shell nanotube arrays on Ti foil substrates is illustrated in Fig. 1. The sacrificial template of Co_2_(OH)_2_CO_3_ nanowire arrays were firstly synthesized on Ti foils through a hydrothermal reaction. TiO_2_ was uniformly deposited on the surface of the nanowire arrays, followed by etching of Co_2_(OH)_2_CO_3_ templates. Finally, an outmost layer of Fe_2_O_3_ was uniformly grown intimately onto the double-side of TiO_2_ nanotube architectures with thickness (mass) controllable synthesis precisely regulated by ALD cycles.

**Figure 2:**
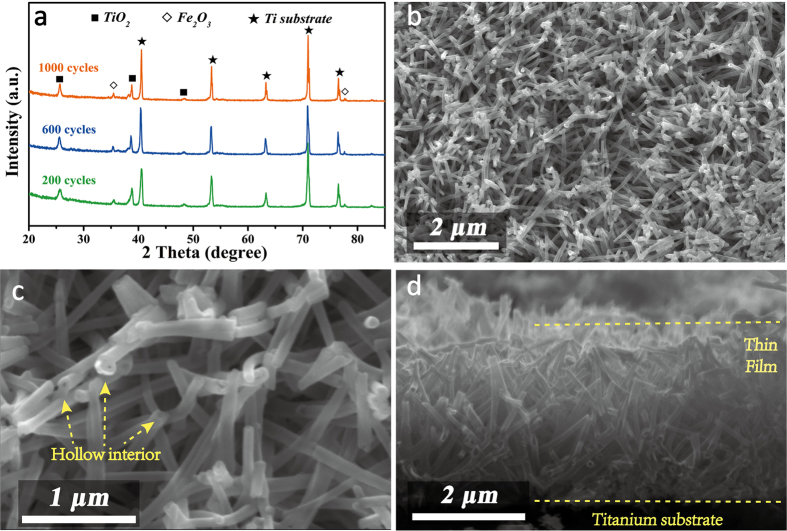
(**a**) XRD patterns of TiO_2_@Fe_2_O_3_ nanotube arrays with different coating loops. (**b**,**c**) SEM images of representative TiO_2_@Fe_2_O_3_-600 sample and (**d**) the cross-section. Hollow interior construction can be detected in (**c**).

The overall crystal structure and phase purity of three samples were characterized by XRD. Shown in Fig. 2a, it is clear that one set of diffraction peaks located at 25.2°, 38.5°, 48.0°, 55.0° and 82.6° correspond to (101), (112), (200), (211) and (224) planes of anatase TiO_2_ (JCPDS no. 21-1272). Additionally, two diffraction peaks at 35.6° and 77.7° with weaker intensity are indexed to (110) and (306) planes of hexagonal α-Fe_2_O_3_ (JCPDS no. 33-0664). Accordingly, Fe_2_O_3_ proportions are calculated as 25.1 wt%, 33.6 wt% and 44.6 wt% for TiO_2_@Fe_2_O_3_ samples by increasing ALD cycles to 200, 600 and 1000 cycles, which is in good accordance with the evolution of diffraction intensity in XRD and Raman spectra (Figure S1). Except for patterns of Ti foil substrate, no impurity peaks were observed in the XRD patterns, affirming the existence of bi-phase oxide crystal structures for the active materials without any by-products.

Field emission scanning electron microscopy (FESEM) was utilized to investigate morphological evolution of bare TiO_2_ to TiO_2_@Fe_2_O_3_ samples (Figure S2a–d). The pure TiO_2_ material exhibits dense and uniform nanotube arrays without agglomeration of large particles, laying good foundation for high mass loading of active materials. After coating of Fe_2_O_3_ onto the surface, these randomly oriented nanotube arrays of TiO_2_@Fe_2_O_3_-600 across each other to form a highly interconnected network (Fig. 2b), which is favorable for the electron conduction across the whole electrode^32^,^33^. Under higher magnification in Fig. 2c, hollow interiors are discerned for these nanotubes. Even after coating of ALD cycles, the tubular architecture could still be maintained, which is clearly shown from open-ended nanotubes. These open structures with inside perforative channels are conductive to the electrolyte infiltration and will largely benefit ion migration during electrochemical process^34^. The overall cross-section image of TiO_2_@Fe_2_O_3_-600 manifests array length of micron order with perfect attachment and connection to Ti substrate (Fig. 2d).

**Figure 3:**
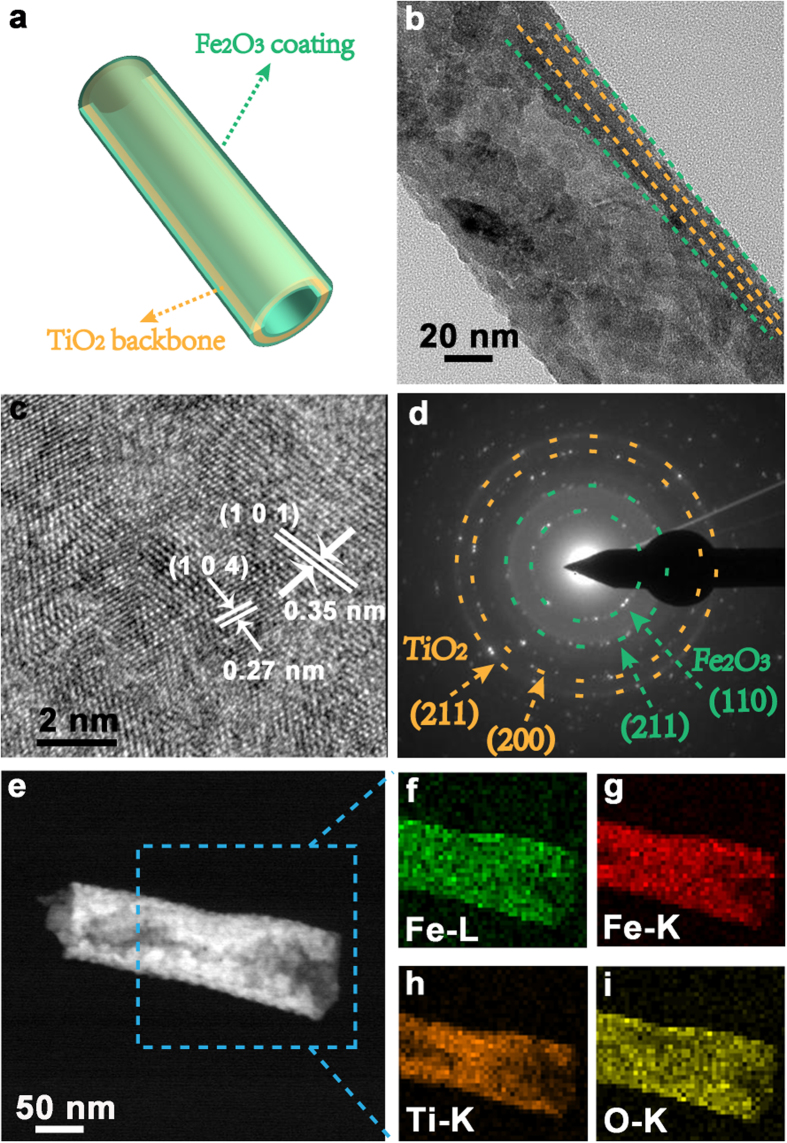
(**a**) Schematic diagram of an individual TiO_2_@Fe_2_O_3_-600 nanotube. (**b**) TEM image and (**c**) high resolution image taken from the fringe of a typical nanotube, and (**d**) corresponding SAED patterns. (**e**) Dark-filed TEM image of a single TiO_2_@Fe_2_O_3_ nanotube and the element mapping (**f**–**i**).

More detailed texture and structure are revealed by transmission electron microscopy (TEM) and schematic illustration of one single TiO_2_@Fe_2_O_3_ nanotube (Fig. 3a), identified with characteristic hollow of the nanotube and with diameter of around 100 nm (Fig. 3b). Further enlarged at the edge in Fig. 3c, high-resolution TEM (HRTEM) images focusing on the hetero-junction region display two distinct sets of lattice fringe spacing as 0.27 and 0.35 nm, matching well with the (104) plane of the hexagonal α-Fe_2_O_3_ and the (101) plane of the anatase TiO_2_, respectively. Corresponding selected area electronic diffraction (SAED, Fig. 3d) pattern further confirms the coherent existence of well-crystallized TiO_2_ and Fe_2_O_3_ structures, suggesting the success building of intimate interaction at the interface. The obviously spotted diffraction rings are in well correspondence with (110) and (211) planes of *α*-Fe_2_O_3_, together with the (211) and (200) planes of anatase TiO_2_, which is consistent with the XRD analysis. Moreover, element mapping of Fig. 3f–i derived from dark-field SEM (Fig. 3e) confirms that all the elements are homogeneously dispersed within the overall nanotube. It is noted that distributions of Fe and O confirm a wrapped picture around the element of Ti, providing another evidence for the Fe_2_O_3_-coated core-shell structure.

**Figure 4:**
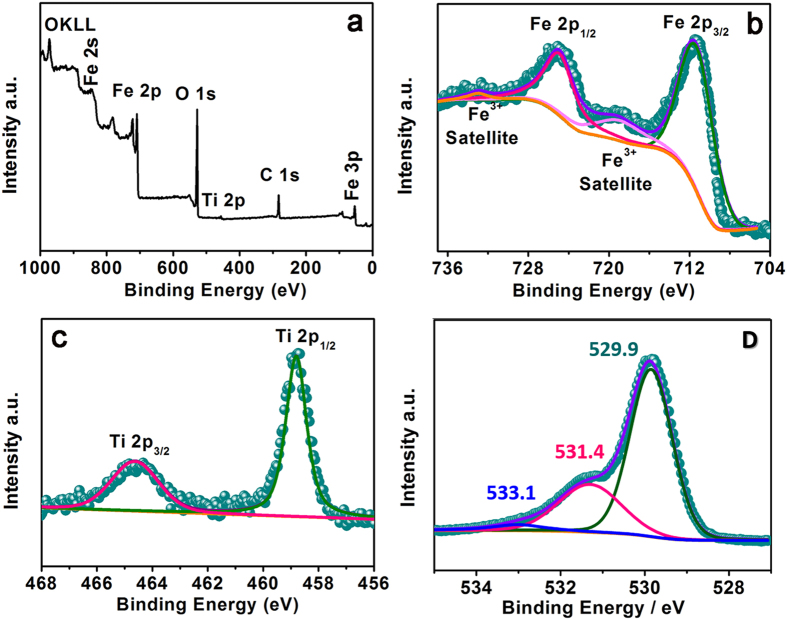
(**a**) XPS spectrum of a wide survey scan for the TiO_2_@Fe_2_O_3_-600 nanotube arrays, and high-resolution peaks of (**b**) Fe 2p, (**c**) Ti 2p, (**d**) O 1 s region.

To determine the chemical compositions and surface bond states of the obtained materials, XPS test was conducted on the representative TiO_2_@Fe_2_O_3_-600 sample. From a wide survey scan in Fig. 4a, peaks of C 1 s, O 1 s, Ti 2p and Fe 2p were detected, which is accordance with the EDS measurement (Figure S3). The C1s peak originates from adventitious carbon^25^. High resolution spectrum of Fe 2p is comprised of two distinct peaks around 711 eV and 724.5 eV (Fig. 4b), which correspond well to the Fe 2p^3/2^ and 2p^1/2^ with satellite lines. The spectrum is consistent with the characteristic of Fe^3+^ in α-Fe_2_O_3_^24^,^25^,^35^, indicating no reductive component consisting of Fe^2+^ were generated within ALD process. As shown in Fig. 4c, the binding energy of Ti 2P^1/2^ and Ti 2P^3/2^ core levels are observed at approximately 458.9 and 464.6 eV with ~6.3 eV peak splitting, confirming Ti^IV^ state in the anatase TiO_2_^23^,^25^. The spectrum of O1s core level is shown in Fig. 4d, where binding energy peaks at 531.4 and 533.1 eV possibly originate from bonded hydroxyl groups and surface absorbent, respectively^36^,^37^. As for the broad peak centered at 529.9 eV, it is attributed to metal-bonding in both oxides.

**Figure 5:**
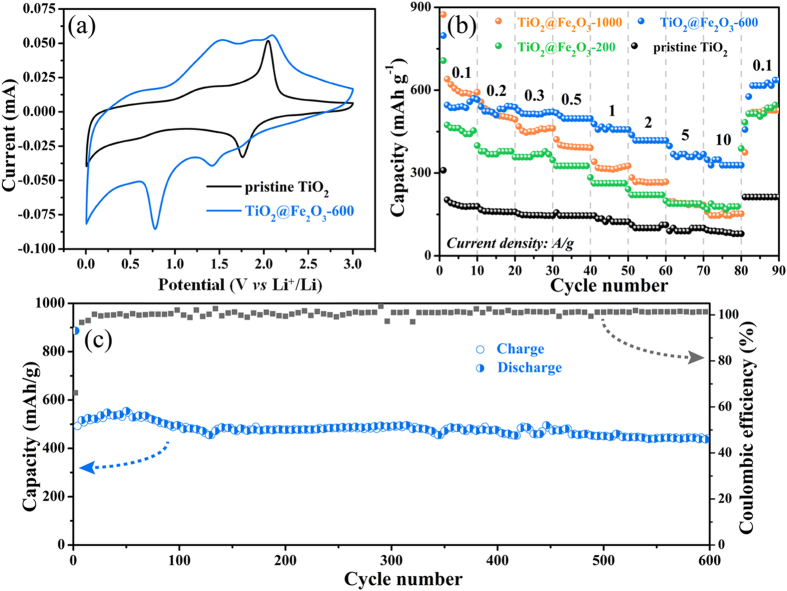
(**a**) CV curves of pristine TiO_2_ and TiO_2_@Fe_2_O_3_-600 samples at a scan rate of 0.5 mV/s (0.005–3 V). (**b**) Rate performance at multiple current densities from 0.1 A/g to 10 A/g then back to 0.1 A/g. (**c**) Capacity vs. cycle number plots and corresponding coulombic efficiency of the TiO_2_@Fe_2_O_3_-600 at a current density of 0.1 A g^−1^.

The cyclic voltammetry (CV) was carried out firstly to explore the electrochemical behavior of the binary oxides structure, while the representative curve is shown in Fig. 5a. During the cathodic scan process, each of the TiO_2_@Fe_2_O_3_ arrays exhibits three reduction peaks around 1.75 V, 1.4 V and 0.8 V. The first peak is ascribed to the phase transition from tetragonal TiO_2_ to orthorhombic Li_x_TiO_2_^23^, which is consistent with the behavior of bare TiO_2_. The second peak is associated with the formation of cubic Li_2_Fe_2_O_3_^38^. The third peak reflects the complete reduction from Fe^2+^ to Fe^0^ as well as generation of amorphous Li_2_O^39^. For the anodic scan, oxidized peaks centered around 1.5–2.5 V correspond to the successively reversible process, including oxidation of Fe^0^ to Fe^3+^, delithiation of Li_x_TiO_2_ as well as conversion of Li_2_O. To investigate the influence of mass balance, TiO_2_ with different amount of Fe_2_O_3_ coating were synthesized and systematically studied. Figure S4 describes the discharge-charge curves for the first cycle at a current density of 0.1 A g^−1^ (0.005–3 V *vs.* Li/Li^+^), wherein the similar voltage plateaus consistent with CV scans are identified (Figure S5). For the bare TiO_2_ nanotubes, the initial discharge and charge capacities are limited to 307 and 173 mA h g^−1^, respectively. By contrast, TiO_2_@Fe_2_O_3_ composites exhibited noticeable improvement. As for TiO_2_@Fe_2_O_3_ samples, the charge capacity raises along with the amount of Fe_2_O_3_ coating. When it comes to TiO_2_@Fe_2_O_3_-1000, the initial discharge and charge capacities can reach up to 878 and 590 mA h g^−1^, with the irreversible capacity loss cut down from 43.7% to 32.8%, which is ascribed to good electrochemical reversibility of *in situ* generated metal nanoparticles. Considering the benign and intimate connection, it is reasonable that the Fe^0^ nanoparticles produced at the interface between TiO_2_ and Fe_2_O_3_ can advance the reversibility of reactions and further result in a high reversible capacity. The TiO_2_@Fe_2_O_3_ hybrid electrodes all show higher capacity than bare TiO_2_ nanotube electrode owing to the introduction of high-capacity Fe_2_O_3_. Particularly shown in Fig. 5c, the TiO_2_@Fe_2_O_3_-600 electrode maintains a high capacity delivery of 436 mA h g^−1^ even after 600 cycles with above 87% capacity retention, which are clearly distinguished from previous reported hybrid TiO_2_-Fe_2_O_3_ materials operated less than 200 cycles^23^,^24^,^25^,^40^. Furthermore, the coulombic efficiency rapidly increases and stays at a high level of around 100% in the subsequent cycles. As revealed in Figure S6, the morphology and texture of the nanotube architecture maintains well after 20 discharging/charging cycles.

For comprehensive understanding of electrochemical performance, rate capability tests were conducted with different current densities from 0.1 A g^−1^ to 10 A g^−1^ (Fig. 5b). It is encouraging that TiO_2_@Fe_2_O_3_-600 sample exhibits outstanding rate performances, with high capacity of ~390 and ~330 mA h g^−1^ even at uprated 5 and 10 A g^−1^, which is more than three-fold of bare TiO_2_ electrodes. Energy storage devices could be served at large currents is highly required for realistic circumstance, while those reported TiO_2_/Fe_2_O_3_ composites are always operated under current densities of 1.6 A g^−1^^23^,^24^,^25^. Such outstanding rate capability of the TiO_2_@Fe_2_O_3_ core- shell nanotube arrays benefits from the unique 1D tubular structure with fast kinetics established from hollow interior. By contrast, although TiO_2_@Fe_2_O_3_-1000 sample demonstrates attracting capacity at a low rate of 0.1 A g^−1^, it drops sharply with escalating currents and is much inferior to TiO_2_@Fe_2_O_3_-600 above the current density of 0.3 A g^−1^.

## Discussion

Overall, high capacity of Fe_2_O_3_ component elevates the holistic lithium storage, while its instability is compensated by the robust scaffold of TiO_2_ nanotubes. These complementary properties may lead to trade-off wherein optimal content are critical yet still need to be precisely studied. Taking advantage of the precisely controllable ALD method, it is found herein that excessive loading of Fe_2_O_3_ will lead to unfavorable attachment with TiO_2_, thus impair the structural stability and electrical conductivity of entire composite along with cycling, though high capacity is obtained at a higher mass loading. With a proper mass loading of 33.6 wt%, TiO_2_@Fe_2_O_3_-600 achieve favorable contact with TiO_2_ substrate while still maintains considerable lithium storage sites, beneficial for the superb fast-stable energy storage balance, which can be regarded as general guidance for materials design of TiO_2_-based binary oxides. Furthermore, after returning to the initial current density of 0.1 A g^−1^, all samples recover back to the original capacity, confirming that the robustness of TiO_2_ arrays matrix in hybrid electrode materials even under high rates circumstance.

To prove the 1D advantage and further understand the optimal Fe_2_O_3_-TiO_2_ balance, the electrochemical impedance spectra are conductive to illuminate kinetics difference (Figure S7). The TiO_2_@ Fe_2_O_3_-600 manifests the fastest ion diffusion than other hybrid materials judging from Warburg impedance values (*Z*_*w*_), demonstrating the efficient ion diffusion within this optimized thickness of coating-wall. It is mentioned that 1D nanotube structure is profitable for the fast kinetics by improving the electrolyte infiltration as well as shorten ion and electron immigration distance, also offering high loading of electrochemical-active sites through double sides coating and mitigation against the volume change of Fe_2_O_3_. Also, optimum mass loading endows TiO_2_@Fe_2_O_3_-600 with the best complementary effect for superb and more stable electrochemical performances.

In this work, binder-additive-free TiO_2_@Fe_2_O_3_ nanotube arrays with double-wall coating are prepared through hydrothermal and controllable ALD methods. In addition to the traditional advantages of 1D structure such as fast transport and good electrolyte contact, the rational design of hollow TiO_2_ tube provides a robust backbone to efficiently hold structural stability and the introduction of double-wall Fe_2_O_3_ coating largely enhance overall energy storage. Particularly, taking advantage of this precisely controllable ALD, an optimal proportion is found to desirably maximize the complementary effects within components, which offers deeper understanding on merits of this binary oxide design. As a result, the hybrid electrode delivers outstanding electrochemical performance in terms of high reversible capacity (520 mA h g^−1^ at 100 mA g^−1^) over three-fold of bare TiO_2_ electrodes and especially long term stability of >87% capacity retention after 600 cycles as well as superb rate capability that could be reversibly operated even at uprated 10 A g^−1^. The presented synthetic techniques of thin-films arrayed electrode are readily extended to other alternative multi-components electrode and are promising for fabricating micro-scale energy storage devices in the future.

## Methods

### Synthesis of Co_2_(OH)_2_CO_3_ nanowire arrays on Ti substrates

Co_2_(OH)_2_CO_3_ nanowire arrays were prepared through a simple hydrothermal method. Briefly, 0.6 g Co(NO_3_)·6H_2_O, 0.15 g NH_4_F and 0.6 g CO(NH_2_)_2_ were dissolved in 70 mL water under magnetic stirring to form homogeneous solution. After cleaning by successive sonication in ethanol, acetone and deionized water, Ti foil substrate was immerged in the above solution and placed into Teflon-lined stainless steel autoclaves. The autoclave was heated at 105 °C for 5 h and then allowed to cool down to room temperature naturally. Then obtained foil was softly rinsed with DI water several times and dried in air at 80 °C.

### Synthesis of hollow TiO_2_@Fe_2_O_3_ core/shell nanotube arrays

Firstly, TiO_2_ was deposited on the as-prepared Co_2_(OH)_2_CO_3_ nanowires using a hot wall ALD system with TiCl_4_ and H_2_O as the Ti and O precursors. Deposition was conducted at 400 °C while flow rates of TiCl_4_ and H_2_O were set as 0.6 cm^3^/pulse and 0.5 cm^3^/pulse, respectively. The processing pressures were ~40 Pa in the deposition steps and 27 Pa in the pump-down steps. Then, the products were immersed into 0.6 M HCl solution for 12 h. Co_2_(OH)_2_CO_3_ was therein removed and hollow TiO_2_ nanotube arrays were obtained. Coating Fe_2_O_3_ to form final core shell structure was realized through further ALD process with water and homoleptic dinuclear irontert-butoxide complex (Fe_2_(O^t^Bu)_6_) as Fe and O precursors, respectively. Controllable syntheses were achieved via different coating loops wherein three samples with 200, 600 and 1000 cycles were conducted at 150 °C, respectively under the same conditions, which are denoted as TiO_2_@Fe_2_O_3_-200, TiO_2_@Fe_2_O_3_-600, TiO_2_@Fe_2_O_3_-1000, respectively.

### Materials characterization

The phase purity and crystal structure of products were characterized by X-ray powder diffraction (XRD, Bruker-AXS D8 Advance, Cu Kα radiation, λ = 0.15418 nm). Morphologies and structures were examined using a field-emission scanning electron microscope (FESEM, Quant 250FEG) equipped with energy dispersive spectrometer (EDS) function, a transmission electron microscope (TEM, FEI Philips CM300 UT/FEG) and a high-resolution transmission electron microscope (HRTEM, JEOL JEM-2010) with energy-dispersive X-ray spectroscopy (EDS). The chemical bonds were analyzed through X-ray photoelectron spectroscopy (XPS, ESCALAB 250Xi).

### Electrochemical Measurements

Electrochemical measurements were carried out on Swagelok cells, assembled in an Ar-filled glove box at room temperature. Pure lithium foils were used as both the counter/reference electrodes and the Ti substrate supported TiO_2_ or TiO_2_@Fe_2_O_3_ (with Fe_2_O_3_ coating deposition of different cycles) were firstly cut into small pieces with the size of 0.5 ∗ 0.5 cm and then directly used as the working electrodes without extra binders or conductive additives. 1 M solution of LiPF_6_ in ethylene carbonate and diethyl carbonate (EC/DEC = 1:1 v/v) was used as the electrolyte. The galvanostatic charge-discharge measurements were performed on LANDCT2001A battery test system at different current densities from 0.1 A g^−1^ to 10 A g^−1^ with a cut-off voltage window of 0.005–3 V. Cyclic voltammograms (CV) were measured at a scan rate of 0.5 mV/s with the same voltage range. Electrochemical impedance spectroscopy (EIS) measurements were recorded in the frequency range of 100 kHz to 10 mHz, with an AC amplitude of 5 mV. Both CV and EIS were conducted on a CHI660D electrochemical workstation.

## Additional Information

**How to cite this article**: Zhong, Y. *et al*. Controllable Synthesis of TiO_2_@Fe_2_O_3_ Core-Shell Nanotube Arrays with Double-Wall Coating as Superb Lithium-Ion Battery Anodes. *Sci. Rep.* **7**, 40927; doi: 10.1038/srep40927 (2017).

**Publisher's note:** Springer Nature remains neutral with regard to jurisdictional claims in published maps and institutional affiliations.

## Supplementary Material

Supporting Information
