# Supplementary material for: Controllable Synthesis of TiO2@Fe2O3 Core-Shell Nanotube Arrays with Double-Wall Coating as Superb Lithium-Ion Battery Anodes
Source: Sci Rep. 2017 Jan 18;7:40927. doi: 10.1038/srep40927 (PMC5241879; doi:10.1038/srep40927)
Supplement: Supporting Information [file srep40927-s1.docx]

**Controllable Synthesis of TiO_2_@Fe_2_O_3_ Core-Shell Nanotube Arrays with Double-Wall Coating for Superb Lithium-Ion Battery Anodes**

Yan Zhong^1^, Yifan Ma^1,2^, Qiubo Guo^1,2^, Jiaqi Liu^1,2^, Yadong Wang^3^, Mei Yang^1,2^*^[[1]](#footnote-1)^**, and Hui Xia^1,2^*^[[2]](#footnote-2)^**

*^1^School of Materials Science and Engineering, Nanjing University of Science and Technology, Xiaolingwei 200, Nanjing 210094, China*

*^2^Herbert Gleiter Institute of Nanoscience, Nanjing University of Science and Technology, Xiaolingwei 200, Nanjing 210094, China*

*^3^School of Engineering, Nanyang Polytechnic, 569830, Singapore*


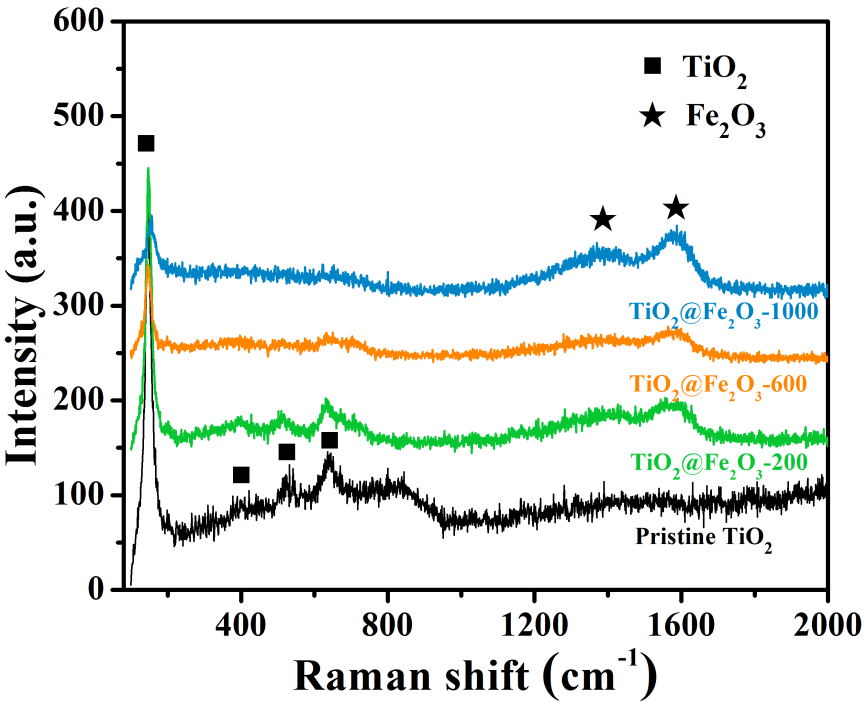


Figure 1. Raman spectra of the pristine TiO_2_ and TiO_2_@Fe_2_O_3_ samples with Fe_2_O_3_ coating deposition of different cycles.


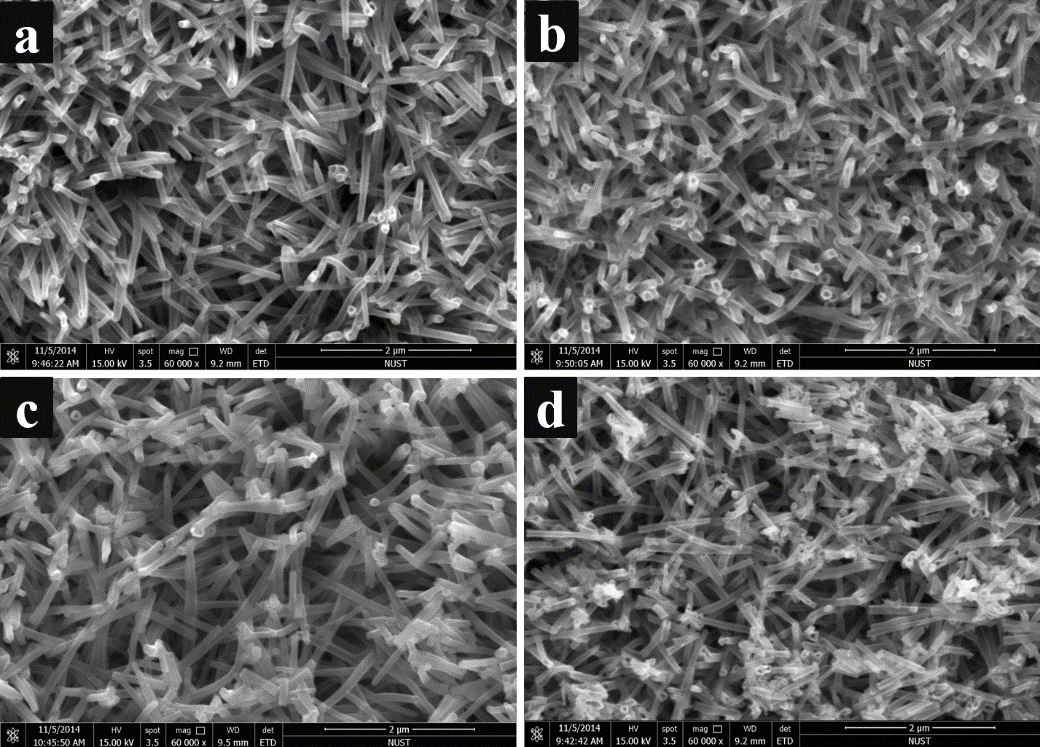


Figure 2. Morphological evolution of bare TiO_2_ to TiO_2_@Fe_2_O_3_ samples: (a) pristine TiO_2_, (b) TiO_2_@Fe_2_O_3_-200, (c) TiO_2_@Fe_2_O_3_-600, (d) TiO_2_@Fe_2_O_3_-1000.


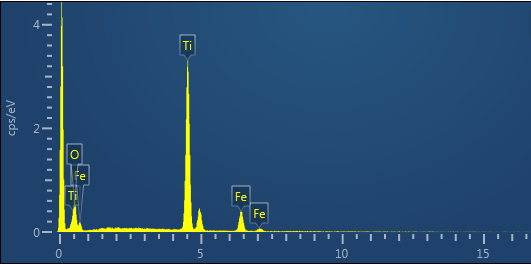


Figure 3. Energy dispersive spectrum (EDS) of the TiO_2_@Fe_2_O_3_-600 sample.


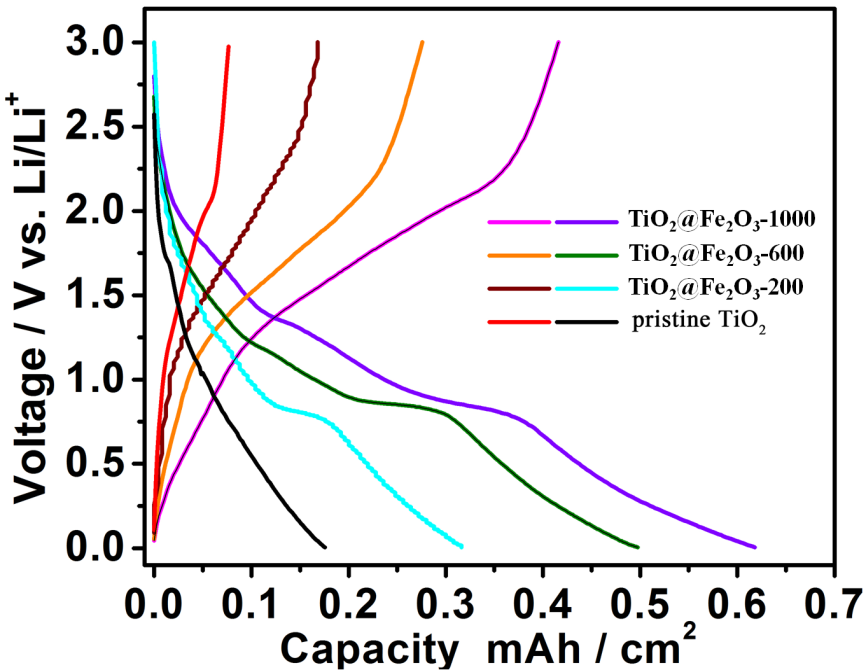


Figure 4. The discharge-charge curves of pristine TiO_2_ and TiO_2_@Fe_2_O_3_ samples for the first cycle at a current density of 0.1 A/g (0.005-3 V *vs.* Li/Li^+^).


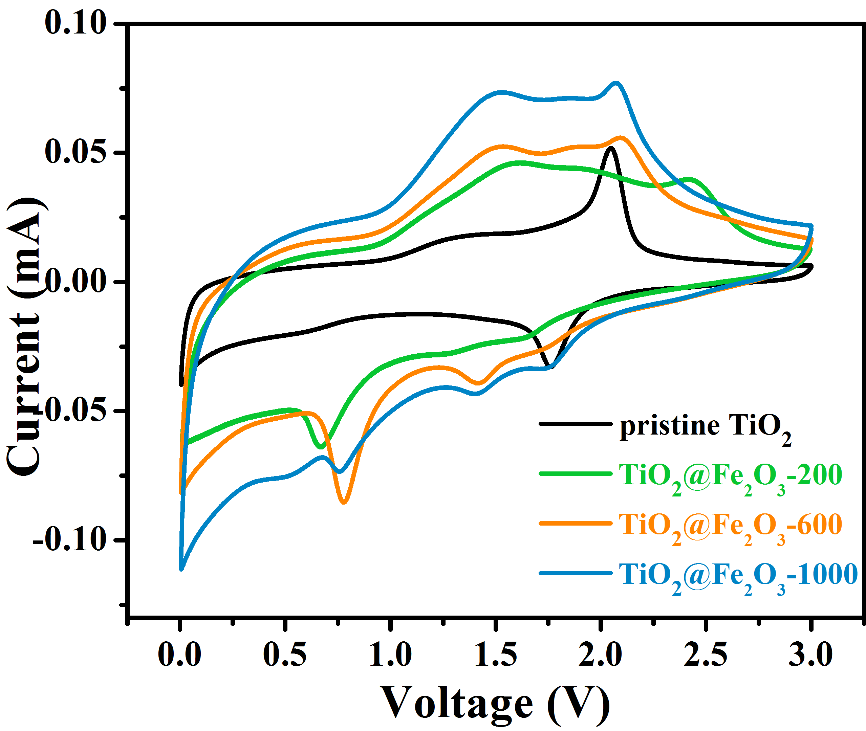


Figure 5. Cyclic voltammetry curves of the pristine TiO_2_ and TiO_2_@Fe_2_O_3_ samples with Fe_2_O_3_ coating deposition of different cycles.


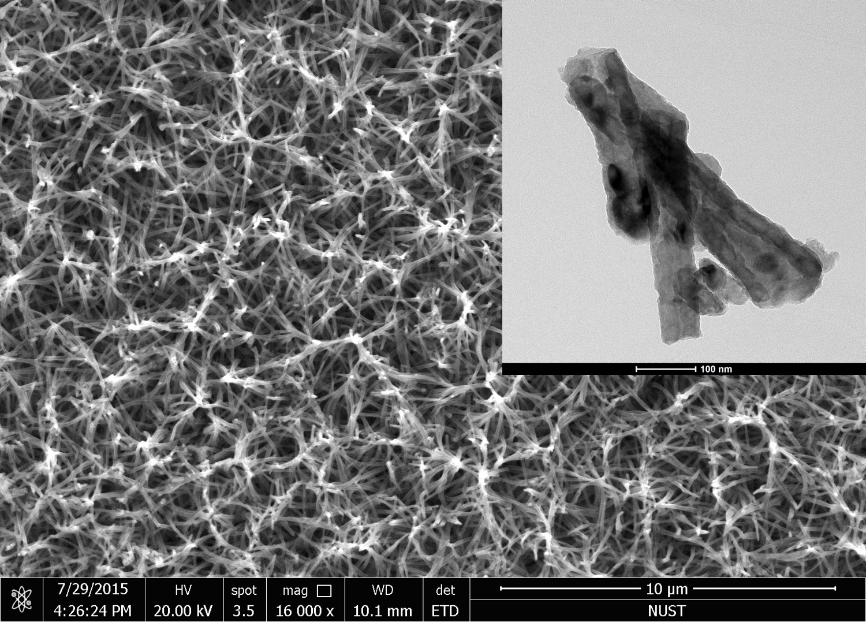


Figure 6. The morphology and texture (inset) of the TiO_2_@Fe_2_O_3_-600 sample after 20 discharging/charging cycles.


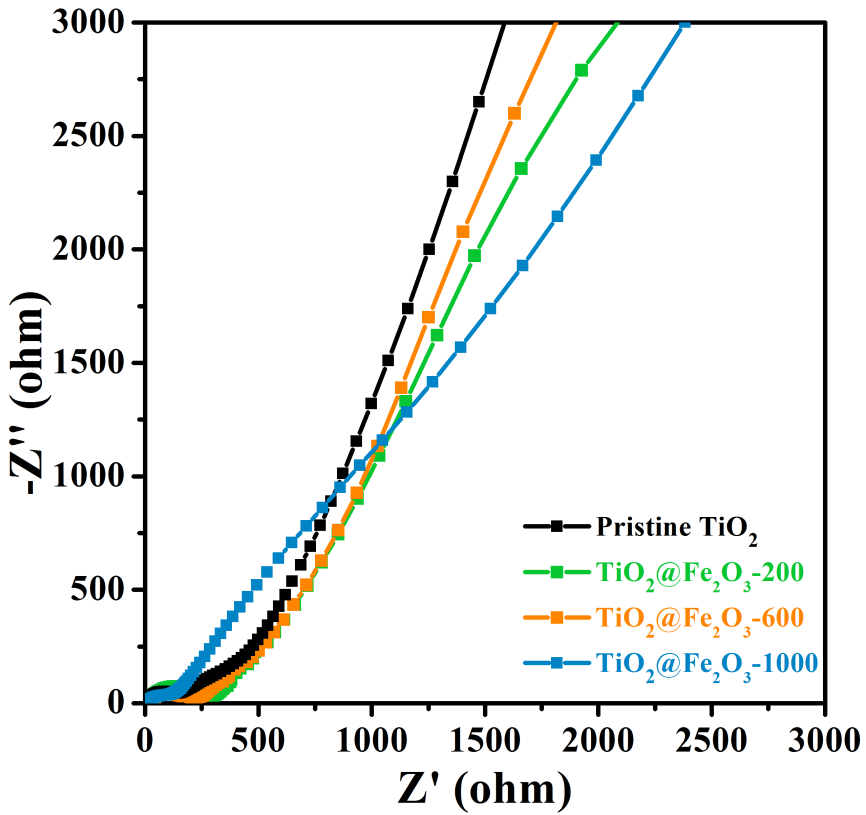


Figure 7. Electrochemical impedance spectra (EIS) of the pristine TiO_2_ and TiO_2_@Fe_2_O_3_ samples.

1. * Corresponding author. Tel./Fax: (86) 25 84303410. E-mail: [bayberry616@njust.edu.cn](mailto:bayberry616@njust.edu.cn) (M. Yang) [↑](#footnote-ref-1)
2. * Corresponding author. Tel./Fax: (86) 25 84303408. E-mail: [xiahui@njust.edu.cn](mailto:xiahui@njust.edu.cn) (H. Xia) [↑](#footnote-ref-2)
